# A Time Off Incentive Was Not Associated with Influenza Vaccination Acceptance among Healthcare Workers

**DOI:** 10.1155/2013/209491

**Published:** 2013-06-26

**Authors:** Saima Cheema, Christopher Vinnard, Sarah Foster-Chang, Darren R. Linkin

**Affiliations:** ^1^University of Tennessee Health Science Center, Memphis, TN 38163, USA; ^2^Division of Infectious Diseases & HIV Medicine, Department of Medicine, Drexel University College of Medicine, Philadelphia, PA 19102, USA; ^3^Philadelphia VA Medical Center, Philadelphia, PA 19104, USA; ^4^Division of Infectious Diseases, Department of Medicine, Center for Clinical Epidemiology and Biostatistics, Perelman School of Medicine, University of Pennsylvania, Philadelphia, PA 19104, USA

## Abstract

*Objectives*. The national influenza vaccination rate among healthcare workers (HCWs) remains low despite clear benefits to patients, coworkers, and families. We sought to evaluate formally the effect of a one-hour time off incentive on attitudes towards influenza vaccination during the 2011-2012 influenza season. *Methods*. All HCWs at the Philadelphia Veterans Affairs (VA) Medical Center were invited to complete an anonymous web-based survey. We described respondents' characteristics and attitudes toward influenza vaccination and determined the relationship of specific attitudes with respondents' acceptance of influenza vaccination, using a 5-point Likert scale. *Results*. We analyzed survey responses from 154 HCWs employed at the Philadelphia VA Medical Center, with a response rate of 8%. Among 121 respondents who reported receiving influenza vaccination, 34 (28%, 95% CI 20–37%) reported agreement with the statement that the time off incentive made a difference in their decision to accept influenza vaccination. *Conclusions*. Our study provides evidence that modest incentives such as one-hour paid time off will be unlikely to promote influenza vaccination rates within medical facilities. More potent interventions that include mandatory vaccination combined with penalties for noncompliance will likely provide the only means to achieve near-universal influenza vaccination among HCWs.

## 1. Background

The Advisory Committee on Immunization Practices (ACIP) and the Healthcare Infection Control Practices Advisory Committee recommend that all HCWs be vaccinated annually against influenza [1]. Vaccination of HCWs against influenza has been shown to prevent transmission of influenza to patients [2, 3], coworkers [4], and families [5]. Despite these benefits, the national influenza vaccination rate among HCWs remains low [6]. Vaccination uptake is limited by concerns regarding vaccine safety and efficacy and misconceptions regarding the perceived risk of nosocomial transmission of influenza [7].

Late in the 2010-2011 influenza season, we instituted a novel incentive to promote influenza vaccination, providing one-hour paid time off immediately following receipt of vaccination, and we achieved our target vaccination rate soon after this incentive program was begun. Based on this experience at our medical facility, we sought to evaluate formally the effect of this time off incentive on attitudes towards influenza vaccination during the 2011-2012 influenza season. We hypothesized that HCWs who had received the influenza vaccine would report a more favorable attitude regarding this incentive (as compared with unvaccinated HCWs). In addition, we sought to identify other attitudes associated with acceptance of influenza vaccination at our medical center.

## 2. Methods

### 2.1. Settings and Participants

The Philadelphia Veterans Affairs (VA) Medical Center is a federal health care facility that includes outpatient clinics, a 145-bed acute care hospital, and an adjoining 240-bed long-term care facility. In our facility, influenza vaccine is provided free of charge by a nurse-staffed roving cart that visits each floor of the facility on several occasions during the influenza season. The target vaccination rate for the facility, established by the national VA administration, was 80% for the 2010-2011 influenza season and 85% for the 2011-2012 influenza season.

### 2.2. Data Collection

All HCWs were invited by email and newsletters to complete an anonymous web-based survey (SurveyMonkey), developed with input from Infection Control Practitioners at the authors' institutions and piloted in a convenience sample of employees at the Philadelphia VA Medical Center across a range of job duties. The survey questions were based on prior CDC work regarding influenza vaccination among HCWs [8]. We excluded survey respondents who indicated that they were not HCWs at our facility during the period of the incentive.

### 2.3. Analysis

We described respondents' characteristics and attitudes toward influenza vaccination, including their agreement or disagreement with the statement, “The one hour of time off award made a difference in my decision whether to accept the flu vaccination.” We then classified survey respondents into two groups: those that accepted or did not accept influenza vaccination during the 2011-2012 influenza season. We determined the relationship of specific attitudes with respondents' acceptance of influenza vaccination, using a 5-point Likert scale (1 “strongly disagree” to 5 “strongly agree”). We used the nonparametric Kruskal-Wallis test to compare Likert responses between the two groups. 

## 3. Results

We obtained 195 survey responses out of 2473 paid employees at the Philadelphia VA Medical Center, corresponding to a response rate of 8%. After excluding 41 respondents who indicated that they were not HCWs at our facility during the 2011-2012 influenza season, we analyzed survey responses from 154 HCWs employed at the Philadelphia VA Medical Center. 132 respondents (86%) reported receiving the influenza vaccine during the 2011-2012 influenza season. Characteristics of survey respondents are shown in Table 1. The overall vaccination rate for HCWs at the medical facility for the 2011-2012 influenza season was 61%.

We compared the Likert scores for respondents with and without influenza vaccination during the 2011-2012 influenza season (Table 2). HCWs that did not receive influenza vaccination during this time period indicated significantly greater concerns regarding the safety and efficacy of the influenza vaccine. Furthermore, these HCWs minimized their personal risk of influenza given their duties as HCWs, compared to respondents that reported a history of vaccination.

A total of 137 respondents indicated their attitude towards the time off intervention, with a mean Likert score of 2.7. Among 121 respondents who reported receiving influenza vaccination, 63 (52%, 95% CI 43–61%) reported disagreement with the statement that the time-off incentive made a difference in their decision to accept influenza vaccination, 24 (20%, 95% CI 13–28%) reported a neutral attitude towards this statement, and 34 (28%, 95% CI 20–37%) reported agreement with this statement.

## 4. Discussion

In our study, the attitudes of HCWs towards the paid time-off incentive were not associated with their decision to accept influenza vaccination. Interventions to improve vaccination rates among HCWs such as educational programs [9–11] and facilitated access [12] have resulted in modest improvements in influenza vaccination rates. Thus far, incentives alone (without other program components) have not been found to improve vaccination rates among HCWs [13].

Similar to prior studies, we found that influenza vaccination acceptance was limited by negative attitudes concerning vaccine efficacy and safety [7, 9]. We also observed that unvaccinated HCWs reported less concern regarding their personal risk of contracting influenza in the healthcare setting. Recent work to understand the factors that motivate influenza vaccination, based on the theory of planned behaviors, suggests that these motivational attitudes are the primary determinants of the intention to be vaccinated against influenza, rather than logistical concerns such as the convenience or cost of vaccination [14].

Our study has several important limitations. Given the more than 2,000 HCWs that are employed at the Philadelphia VA Medical Center, our response rate was small, and the anonymous nature of the survey did not permit us to compare respondents and nonrespondents, leading to the potential for sampling bias. This potential bias is particularly important given the small number of respondents who reported not receiving the seasonal influenza vaccine during the 2011-2012 season. Because of the anonymous nature of the survey, influenza vaccination status was based on self-report. Finally, we did not assess vaccination status during the preceding (2010-2011) influenza season, which has been shown to be the strongest predictor of acceptance of vaccination during subsequent seasons.

Despite these limitations, our study provides evidence that modest incentives such as one-hour paid time off will be unlikely to promote influenza vaccination rates within medical facilities. More potent interventions that include mandatory vaccination [15] combined with penalties for noncompliance [16] will likely provide the only means to achieve near-universal influenza vaccination among HCWs.

## Figures and Tables

**Table 1:** Characteristics of survey respondents.

Characteristic (total respondents)	Number of respondents (%)
Age categories (154)	
<50	63 (41%)
50–64	72 (47%)
>64	16 (10%)
Prefer not to answer	3 (2%)
Sex (152)	
Male	94 (62%)
Female	58 (38%)
Race/ethnicity (154)	
Black	27 (18%)
White	99 (64%)
Asian	9 (6%)
Hispanic	4 (3%)
Multiple races	4 (3%)
Prefer not to answer	11 (7%)
Occupation (154)	
Facilities	11 (7%)
Allied health	32 (21%)
Administration	42 (27%)
LPN	6 (4%)
RN	32 (21%)
Physician	18 (12%)
Prefer not to answer	13 (8%)

**Table 2:** Attitudes of HCWs regarding influenza vaccination.

Attitude	Mean Likertscore(SD)		*P* value*
	Vaccinated (*n* = 132)	Not vaccinated (*n* = 22)	
The flu vaccine can cause the flu.	1.7	2.3	0.02
The flu vaccine will make me sick.	1.9	2.9	<0.01
I do not get the flu.	2.2	3.2	<0.01
If I get the flu vaccine it protects me against getting the flu.	4.1	3.3	<0.01
If I get the flu vaccine it protects patients I may interact with against the flu.	4.2	3.5	<0.01
If I get the flu vaccine it protects family members or others I am close to against the flu.	4.1	3.5	0.01
Healthcare workers are at increased risk of getting the flu.	4.5	3.9	0.01
Flu vaccination is convenient at work.	4.8	4.6	0.46
I do not like getting shots/needles.	3.1	3.7	0.06
Flu is not a serious illness.	1.7	1.7	0.48

*Kruskal, Wallis equality of populations rank test.
